# Colorectal tumors with complete obstruction – Endoscopic recovery of passage replacing emergency surgery? A report of two cases

**DOI:** 10.1186/1471-230X-7-14

**Published:** 2007-03-28

**Authors:** Giuliano Ramadori, Alexander Lindhorst, Thomas Armbrust

**Affiliations:** 1 Department of Gastroenterology and Endocrinology, Georg-August-University of Göttingen, Germany

## Abstract

**Background:**

Incomplete or complete obstructive ileus due to colorectal cancer is generally treated by emergency surgery that has higher morbidity and mortality than elective surgery.

**Case Presentation:**

Here we describe an endoscopic technique by which a safe bowel decompression was performed instead of emergency surgery in two patients with complete tumorous obstruction of the colon. By means of a polypectomy snare, a soft wire, an ERCP catheter, a set of endoscopes with different diameters (baby endoscope, gastroscope) and of argon plasma coagulation the tumor mass was reduced and the tumor stenosis was passed. The patients recovered from symptoms of colon obstruction, no procedure-associated complications were observed. One patient had surgery of the sigmoid tumor one week later (UICC-stage III), the other patient (UICC-stage IV) received systemic chemotherapy starting one week after endoscopic decompression.

**Conclusion:**

Complete tumorous obstruction of the colon may be managed by endoscopic tumor debulking avoiding high risk emergency surgery and allowing immediate medical treatment of the primary tumor and of metastases.

## Background

Colorectal cancer (CRC) ranks among the most common malignancies in the Western World [1]. Although effective screening and risk management is available up to 30% of patients present with locally advanced disease and/or synchronous metastases [2]. The primary tumor may cause symptoms like bowel obstruction and/or colorectal bleeding recognized both by patients and by physicians as emergency situation. In fact, in up to 38% of patients presenting with large bowel obstruction CRC is diagnosed [3]. Management of tumorous obstruction of the colon by CRC generally consists in surgical colostomy or ileostomy. Resection of the tumor as part of a two-stage procedure after the patient has recovered from acute obstruction still is frequently performed even in stage IV CRC in surgical clinics although a benefit in survival has to be questioned. We recently described a small series of patients with stage IV CRC in whom the non-obstructive primary was treated endoscopically. Highly effective systemic chemotherapy had been applied immediately after endoscopic management of the primary and this policy may have avoided subsequent colon obstruction by the primary [4]. Now we describe two patients who presented with symptoms of complete large bowel obstruction who underwent a successful endoscopic recanalization.

## Case Presentation

### Patient 1

**Figure 1:**
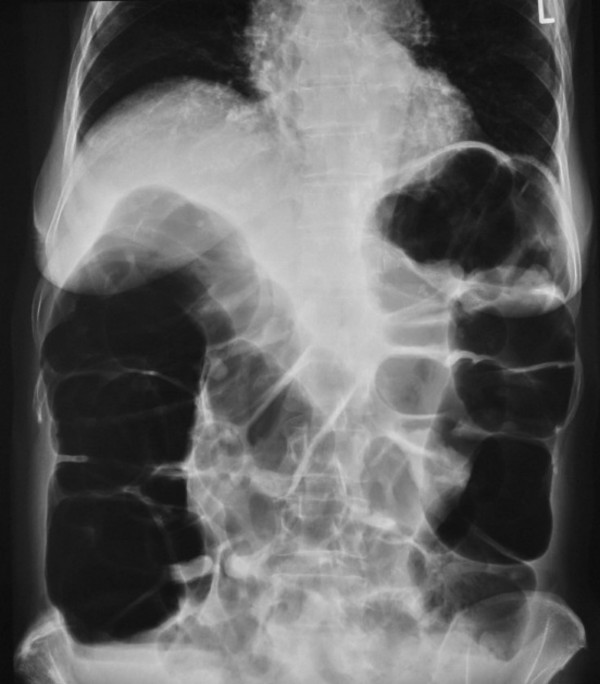
Abdominal X-ray of patient 1 at first presentation. Note heavily dilated colon frame.

**Figure 2:**
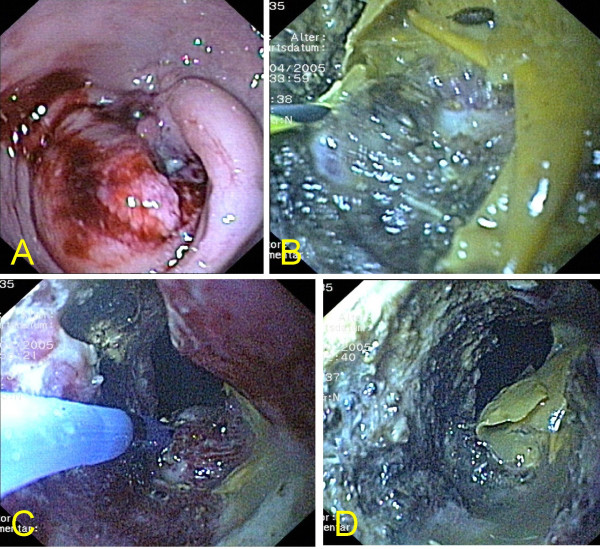
Colonoscopic picture of patient 1 at first presentation showing stenosis of the sigmoid which could not be passed by the colonoscope (A). Endoscopic snare debulking of the distal part of the tumor allowed to push the wire beyond the stenosis (B). After passing the tumor APC ablation of the proximal part of the tumor could be performed opening a channel (C). View of the stenosis after complete debulking. Note the channel with stool covering the luminal surface (D).

A female patient born in 1930 was admitted to our hospital due to sustained abdominal pain and obstipation. X-ray of the abdomen revealed heavy dilatation of the colon and small intestine suggesting an obstruction of the distal colon (figure 1). The general condition of the patient was moderately reduced. She refused a surgical treatment, but aggreed to have a colonoscopy that was performed without prior fluoroscopy. Colonoscopy performed with a regular colonoscope (Olympus Q 145L colonoscope) showed a tumorous obstruction of the sigmoid (figure 2A). The distal parts of the tumor close to the endoscope were removed using a polypectomy snare. A baby endoscope instead of the colonoscope (Olympus XP160 gastroscope, 6 mm diameter) was then placed near to the tumor and a soft wire (Boston Scientific Jagwire, 450 cm length, 0.89 mm diameter) was placed beyond the stenosis. The endoscope was pushed forward until the proximal end of the tumor was reached and the normal lumen of the colon could be identified. The endoscope was then withdrawn leaving behind the wire. An Endoflex ERCP catheter (2.3 mm/1.8 mm) was pushed forward over the wire beyond the stenosis and used as a guide to introduce a conventional gastroscope (Olympus Q145 gastroscope, 10 mm diameter). This was pushed forward guided by the catheter. Due to the larger diameter of its working channel an argon plasma coagulation probe (APC probe from Erbe Elektromedizin, Tübingen, Germany) with side fire then could be used. By APC during stepwise retraction of the endoscope a channel was generated (figure 2C). The new lumen was wide enough to allow the passage of the colonoscope at the following day (figure 2D) and further debulking could be performed with the snare. No procedure-induced symptoms were observed. Abdominal ultrasound revealed diffuse, non-resectable liver metastases and chemotherapy including oxaliplatin, 5-fluorouracil and folinic acid was started 7 days after the initial debulking. The patient had regular bowel movements again and bowel obstruction did not occur anymore. No complications that were caused by the primary could be observed thereafter. The patient died one year later because of liver failure due to progressive liver metastases in spite of switching to second line chemotherapy.

### Patient 2

**Figure 3:**
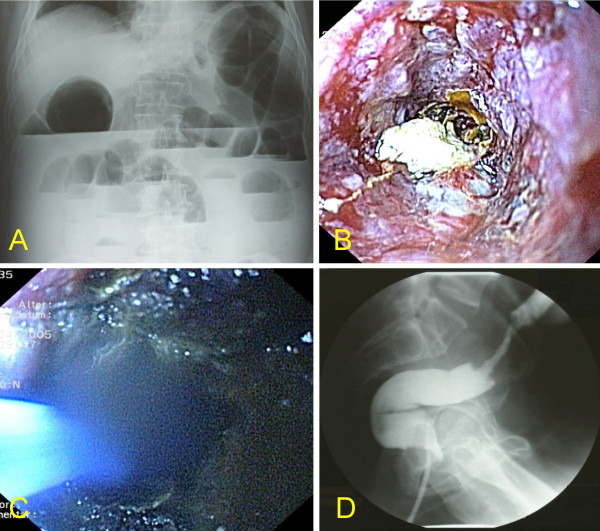
Abdominal X-ray of patient 2 at the time of first presentation showing complete colon obstruction and dilatation (A). Colonoscopic view of the sigmoid tumor with complete obstruction (B). Colonoscopic view during APC ablation of the tumor (C); X-ray enema two days after APC ablation showing the channel through the stenosis (D).

A male patient born in 1934 without a medical history was brought to our clinics because of obstipation with abdominal pain. His general condition was moderately affected. Abdominal X-ray suggested complete distal obstruction with massive dilatation of the colon (figure 3A). A complete stenosis was found by colonoscopy at 18 cm proximal to the anus (figure 3B). Tumor mass reduction was performed using a polypectomy snare. To pass the tumor the procedure described in case 1 was applied after replacing the colonoscope by the baby gastroscope (figure 3C). The patient recovered from obstructive ileus and had regular bowel movements. An X-ray enema using a water-soluble contrast medium was performed to exclude perforation and to localize the tumor (figure 3D). Staging was performed and since no distant metastases were detected the tumor was surgically resected one week later (pT3N2M0). Accordingly, adjuvant therapy with oxaliplatin, 5-fluorouracil and folinic acid was started after recovery of the patient from surgery.

## Conclusion

In the Western World acute obstruction of the large bowel is most frequently caused by colorectal cancer [5]. Main symptoms are abdominal pain, acute meteorism, spasms, vomiting. The symptoms may proceed to those of peritonitis and of hypovolemic/toxic shock, if diagnosis is delayed. It is generally managed as an acute emergency and patients receive extensive diagnostic procedures including abdominal ultrasound, abdominal X-ray, computertomography including intravenous contrast medium administration and even angiographic procedures. In case of a tumorous obstruction which in most patients is located in the left colon [6] surgical procedures may be one-staged (tumor resection and primary anastomosis) or two-staged (colostomy and secondary resection of the tumor). However, there is still no consensus which procedure should be preferred because of high morbidity and mortality associated with each procedure [3]. In fact, a large cohort study has shown that the majority of patients presenting in stage IV CRC receive resection of the primary, but only a minority of them receive chemotherapy within 4 months after surgery [7]. Thirty-days mortality was as high as 10% and it may be concluded that morbidity which is likely to be the main reason precluding chemotherapy was in the range reported earlier [8]. Non-operative management of patients has shown that complication rates due to the primary are lower than expected not justifying preventive resections [9,10].

**Figure 4:**
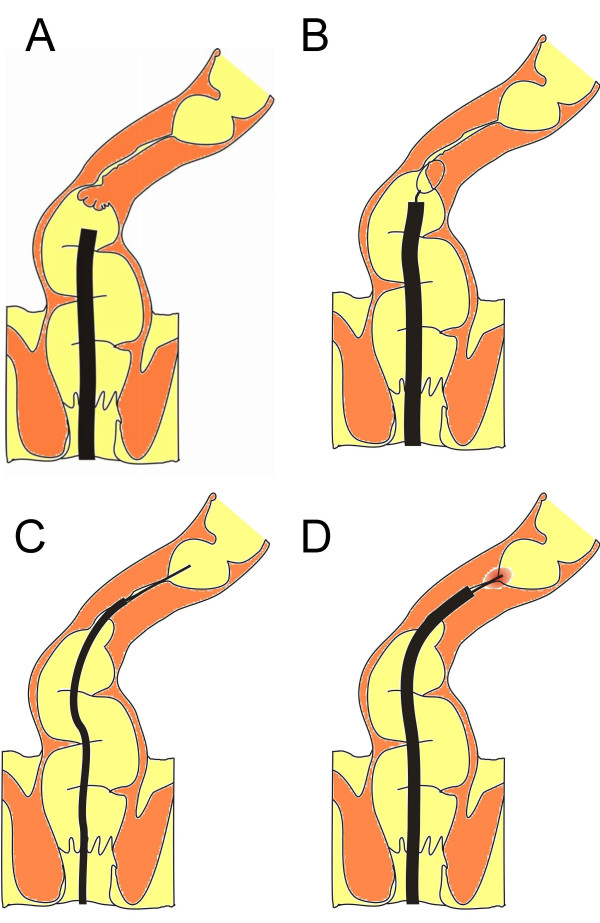
Schematic presentation of first-step endoscopic tumor debulking. A tumorous obstruction of the distal sigmoid colon that cannot be passed with the colonoscope. Polypoid parts of the tumor are removed with the snare (B), and after replacing the colonoscope by a baby endoscope, first a soft wire and then, guided by the wire, the baby gastroscope is pushed through the stenosis (C), the scope is withdrawn, leaving the wire in place, an ERCP catheter is pushed over the wire to stabilize it, and finally a conventional gastroscope that has a working channel wide enough to use a APC probe is pushed beyond the stenosis via the ERCP catheter and the wire. Thereby, APC ablation of the tumor may be performed from the proximal part creating a channel through the tumor (D).

**Figure 5:**
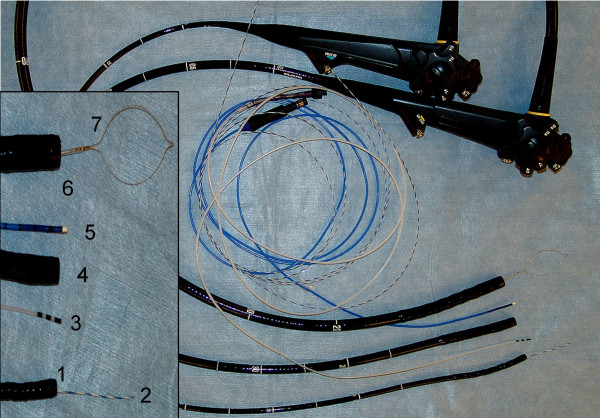
Endoscopes and other tools used for recanalization of tumor stenosis: 1: Olympus XP160 baby gastroscope, diameter 6 mm; 2: Boston Scientific Jagwire guidewire 0.89 mm 3: Erbe APC side fire probe 2200SW 2,3 mm 4: Olympus Q145 gastroscope, 10 mm diameter 5: Endoflex ERCP catheter 2.3 mm 6: Olympus Q145L colonoscope, diameter 14 mm 7: Uno Loop electric polypectomy loop 2.3 mm.

Here we describe an endoscopic procedure that may help to overcome the emergency situation of acute malignant colon obstruction. Primary tumor debulking using a polypectomy snare followed by insertion of a soft wire through the tumor and of a baby endoscope may be performed in any part of the large intestine with low risk for tumor perforation. A conventional gastroscope can then be introduced and further tumor destruction can be achieved by application of argon beamer plasma coagulation (figure 4 and 5). To our experience this particular step is very helpful to overcome acute abdominal complaints caused by air-filled and often highly stretched gut walls. The air can be eliminated through the gastroscope. The procedure allows control of the acute clinical situation eliminating the abdominal discomfort winning time for the patients to recover and for physicians to perform further staging investigations. This is of clinical importance if we consider that more than 50% of patients are older than 65 years. Colonoscopy with tumor debulking, wire-guided passage of the tumor and APC tumor destruction may provide a safe alternative to "salvage" surgery and may reduce morbidity and mortality in a way similar to introduction of metal stents [10,11]. Although metal stents may be an alternative they are more expensive, not always readily available and may dislocate and cause bowel perforation [12]. This may happen more often if we consider that chemotherapy is nowadays more effective than it was before. In stage IV colorectal cancer surgery may be avoided and todays highly effective chemotherapy may be started without delay [4]. We think that a prospective study should be started.

Taken together, complete tumorous obstruction of the colon may be managed by endoscopic tumor debulking as described here. We believe that our technical procedure is safe and it can avoid emergency surgery. Further experience must be collected and the impact on survival of these patients should be investigated.

## Competing interests

The author(s) declare that they have no competing interests.

## Authors' contributions

GR, AL and TA performed the technical procedures described in the manuscript, AL collected the data and arranged the figures and discussed the literature, TA and GR prepared the manuscript. All authors read and approved the final manuscript.

## Pre-publication history

The pre-publication history for this paper can be accessed here:
